# Balancing B cell responses to the allograft: implications for vaccination

**DOI:** 10.3389/fimmu.2022.948379

**Published:** 2022-07-27

**Authors:** Clarkson Crane, Lauren Loop, Christine Anterasian, Bob Geng, Elizabeth Ingulli

**Affiliations:** ^1^ Department of Pediatrics, Division of Pediatric Nephrology, University of California at San Diego and Rady Children’s Hospital, San Diego, CA, United States; ^2^ Department of Pediatrics, Division of Allergy and Immunology, University of California at San Diego and Rady Children’s Hospital, San Diego, CA, United States; ^3^ Department of Pediatrics, Division of Pediatric Infectious Diseases, University of Washington and Seattle Children's Hospital, Seattle, WA, United States

**Keywords:** B cells, antibody mediated rejection, vaccinations, kidney transplant recipients, SARS CoV2 mRNA

## Abstract

Balancing enough immunosuppression to prevent allograft rejection and yet maintaining an intact immune system to respond to vaccinations, eliminate invading pathogens or cancer cells is an ongoing challenge to transplant physicians. Antibody mediated allograft rejection remains problematic in kidney transplantation and is the most common cause of graft loss despite current immunosuppressive therapies. The goal of immunosuppressive therapies is to prevent graft rejection; however, they prevent optimal vaccine responses as well. At the center of acute and chronic antibody mediated rejection and vaccine responses is the B lymphocyte. This review will highlight the role of B cells in alloimmune responses including the dependency on T cells for antibody production. We will discuss the need to improve vaccination rates in transplant recipients and present data on B cell populations and SARS-CoV-2 vaccine response rates in pediatric kidney transplant recipients.

## Introduction

The role of pre-existing humoral antibodies against donor cells mediating immediate graft loss or hyperacute rejection was identified early in the history of transplantation (1). Genotyping human leukocyte antigen (HLA) class I and class II alleles in donors and recipients and careful testing of recipients’ serum for anti-donor HLA antibodies before transplantation to avoid transplanting donors to recipients with preformed antibodies have essentially eliminated hyperacute rejection (2). However, development of anti-T cell responses and *de novo* anti-donor antibodies to allografts remains problematic. Acute allograft rejection is strongly related to the development of biopsy-proven chronic allograft dysfunction and subsequent graft loss (3). Preventing T cell mediated, and antibody-mediated destruction of allografts is the goal of induction and maintenance immunosuppression. However, the intensity of immunosuppression is tempered by the risks of overwhelming infections and post-transplant lymphoproliferative disease.

## The basics: T cell activation

The immune system is comprised to two components—innate and adaptive immunity. The innate system involves natural killer (NK) cells, macrophages, and dendritic cells (DC), among others, that express receptors for a broad range of pathogen- or danger-associated molecular patterns (PAMPs and DAMPs) (4). Once thought to be minimally involved in transplant rejection, it is now recognized that innate system activation can set the stage for an adaptive alloimmune response. This is particularly relevant in that ischemia reperfusion injury (IRI) is thought to incite an innate response that can prime later allograft rejection (5). While this is an area of active study, a detailed discussion of the innate immune system involvement in allograft rejection is beyond the scope of this review.

In light of this, the adaptive immune response is the most well characterized aspect of transplant rejection. Adaptive immune responses are classified as cellular or humoral. Cellular responses are mediated by T lymphocytes while humoral responses are characterized by the production of antibodies by B lymphocytes and plasma cells. Both responses are intertwined and overlap. CD4 T cells provide critical help to B cells to produce antibodies and B cells can play a role in T cell activation *via* antigen presentation, co-stimulation and cytokine secretion. In addition, both T cells and B cells have regulatory functions. The interaction between dendritic cells, naïve T cells, and B cells is critical for the initiation and differentiation of the immune response resulting in elimination of the invading pathogen and induction of a life-long memory response (6).

Naïve lymphocytes circulate between the blood, lymph nodes and spleen. Within secondary lymphoid organs, naïve T and B cells are physically separated (7). Dendritic cells reside within the T cell areas of secondary lymphoid tissue and are the most potent antigen presenting cells (8). They extend their dendrites and are constantly contacting and scanning the surrounding T cells in search of those that are specific for the antigens they have processed and presented on the surface as peptide/major histocompatibility complexes (9). The 3-signal model of T-cell activation in the development of an adaptive immune response applies to allograft rejection (10–13).

Alloantigen recognition by T cells (signal 1) can occur through three different pathways: the direct pathway, the indirect pathway or the semidirect pathway (14–21). The direct pathway occurs when the T-cell receptor on the surface of the T cell interacts with intact allogeneic HLA molecules on the surface of donor antigen-presenting cells (APCs) that have migrated out of the graft to secondary lymphoid tissue. The indirect pathway occurs when donor HLA proteins or other antigens are processed, and peptides are loaded onto recipient MHC complexes and presented on the surface of recipient APCs. The semidirect pathway occurs when recipient APCs acquire and present intact donor-derived HLA molecules. Although it is typically presumed that early acute graft rejection is mediated by the direct pathway while the indirect pathway is responsible for later chronic allograft rejection, this is likely an oversimplification as the indirect pathway has a nuanced involvement in both processes (14).

The second costimulatory signal between APCs and T cell is crucial for productive T cell activation (22). The most extensively studied of the costimulatory signals is CD28 (23). Binding of CD28 to its ligands (B7-1, CD80 and B7-2, CD86) on APCs promotes optimal T-cell receptor (TCR) signaling events that trigger IL-2 production, clonal expansion and generation of effector and memory T cells (24). Inflammation initiates a cascade of events that results in dendritic cell maturation as well as tight and prolonged interactions between antigen bearing DCs and antigen specific T cells whereby costimulatory signals are provided to the T cell (signal 2) resulting in T cell activation and clonal expansion (25). Many other costimulatory molecules have been shown to play a role in T cell activation and there are ongoing efforts underway to block costimulation with the hope of inducing long lasting tolerance (26).

After activation, CD4 T cells interact with CD8 T cells to provide help and the necessary proinflammatory cytokines (signal 3) to promote differentiation into effector and memory cytotoxic T cells and graft rejection (13). Activated CD4 and CD8 T cells alter their cell surface phenotype to express different cell surface molecules enabling cells to migrate from secondary lymphoid organs into peripheral tissues such as the allograft, inducing graft destruction (27). Acute T cell mediated rejection, according to the Banff classification of kidney transplant biopsies, is diagnosed with the presence of interstitial lymphocytic inflammation involving >25% of non-sclerotic cortical parenchyma and tubulitis involving one or more tubules (28).

## B cell activation

Although T cells are sufficient to mediate graft destruction, they play a key role in activating B cells. With the help of CD4 T lymphocytes, B cells are able to generate long-lasting, high-affinity IgG antibodies (29). Within secondary lymphoid tissue, activated antigen specific CD4 T cells migrate to the edges of the T cell zones to interact with antigen specific B cells (30, 31). This interaction is critical for B cell expansion, isotype switching and antibody production (32). When present on the surface of B lymphocytes, the immunoglobulin serves as the antigen receptor for B cells. B cells that bind antigen *via* their B cell receptor upregulate chemokine receptor 7 (CCR7) and Epstein-Barr virus–induced receptor 2 (EBI2) levels on their cell surface and are able to migrate to the B-T cell border (33). The B cells internalize the protein antigen and process it into peptides loaded on class II major histocompatibility complex (MHC) molecules. It is at the T-B cell border where they encounter cognate T cells that provide the necessary costimulatory help. B cells characterized by the expression of the B cell lymphoma 6 (BCL6) transcription factor migrate to the germinal centers and undergo clonal expansion, somatic hypermutation and immunoglobulin isotype class switch recombination, resulting in affinity selection to generate a highly specific anti-donor response (34). The germinal center reaction results in the generation of memory B cells and long-lived plasma cells, both of which allow for a long-term donor-specific humoral immune response (35).

## B cell memory

Most memory B cells bind their specific antigen with a higher affinity than their naïve counterparts (36). Upon antigen re-challenge, memory B cells undergo activation, clonal expansion and germinal center formation much faster and to a more enhanced degree compared to naïve B cells. The result is a rapidly generated high affinity and specific antibody. The longevity of memory B cells is dictated by the BCR signaling and thus will influence the secondary response (37–39). The mechanism underlying control of memory B cell longevity is not well elucidated. A diversity of the BCR occurs as a result of somatic hypermutation (SHM) and class-switch recombination. By uncoupling these two processes, it has been demonstrated that class switching to IgG1 favors the formation of plasma cells while SHM can reduce the longevity of memory B cells (37). Similarly, it has also been suggested that B cell memory relies on varying numbers of isotype switched immunoglobulins and that antigen specific memory B cell longevity may also be a result of genetic predisposition (38, 39). Studies to identify antigen specific B cells in circulation of transplant recipients would be a valuable tool to determine who is at risk for chronic antibody-mediated rejection especially in those cases where donor specific antibodies (DSAs) are not present in circulation (40).

Long lasting plasma cells are also part of the memory response and they persist for several years or even lifelong in the bone marrow (41). They constitutively secrete antibody even in the absence of antigen (42). When directed against the allograft, these secondary responses are problematic. The reactivation of memory B cell responses and generation of long-lived antibody producing plasma cells cause a long-lasting humoral anti-donor immune response resulting in continuous inflammatory damage to the graft that ultimately leads to graft loss. Current immunosuppressive medications are T cell centric and focus on preventing T cell activation. They are very effective at preventing naïve responses but less effective in blunting a memory immune response. By preventing T cell activation, B cell responses and antibody production are thwarted in most cases. However, T cell independent B cell responses have the capability to occur despite adequate maintenance immunosuppression (43). This suggests that some B cell mediated allograft injury and DSA formation can occur in the absence of T cell help.

## Antibody mediated allograft rejection

The hallmark of acute and chronic antibody mediated rejection (AMR) is presence of circulating DSAs, evidence for antibody binding to the vascular endothelium inducing complement activation (e.g., C4d deposition) and microvascular inflammation (44, 45). Antibody producing cells can be short lived while circulating in the blood and long lived when found in bone marrow, secondary lymphoid tissue and peripheral tissues such as an allograft (46). Studies are currently underway to phenotype the various DSA producing cell populations. DSAs can cause a progressive ischemic insult to the allograft eventually resulting in graft loss (45). Five isotypes of antibodies (IgM, IgD, IgG, IgA, and IgE) exist in circulation and are classified according to the constant regions of the heavy chain. The constant domains make up the Fc fragment, which mediates effector functions *via* Fc receptors and complement activation. The variable domains of the antibodies determine antigen specificity and antigen binding. The antibodies that are involved in allograft rejection are usually IgG (47). There are four subclasses of IgG antibodies (IgG1, IgG2, IgG3, and IgG4) with IgG1 being the predominant subclass in circulation. Each isotype varies in its half-life, ability to cross placenta, the degree to which it can neutralize pathogens, and activate macrophages or complement. The nature of the antigen and the cytokine environment during activation results in the different isotypes. Various subclasses of donor specific anti-HLA antibodies have been described in transplant recipients (23, 24, 48) and appear to have the same distribution in the plasma as the general immunoglobulin population.

DSAs can be directed again HLA and non-HLA molecules and have been reported to be associated with poor renal allograft outcomes (49–51). The presence of circulating DSA is a biomarker for T cell activation and previous acute or chronic cellular rejection (52–55). DSAs bind to endothelium of the allograft and have the potential to cause microvascular inflammation, ischemia and graft damage (i.e., glomerulitis, and peritubular capillaritis) (28, 56).

DSA can be of the immunoglobulin subclasses that can fix complement and those that cannot. The binding of DSA to the endothelial cell surface activates the classical complement pathway by engaging the C1 complex. The membrane attack complex is generated and the coagulation cascade is initiated resulting in thrombosis, fibrinoid necrosis, ischemia and loss of graft function (47). As a covalently bound degradation product of the classical complement pathway, C4d deposits detected on the endothelium serve as a marker of AMR (57). In particular, it has been shown that C1q binding DSA portend a higher risk of adverse graft outcomes compared to non-C1q binding DSA (55). Alternatively, circulating DSA that do not fix complement promote graft damage by engaging the Fcγ receptors on natural killer cells, macrophages and neutrophils (58) resulting in the release of growth factors, endothelial and smooth muscle cell proliferation or platelet activation (59, 60).

Chronic AMR has emerged as a leading cause of kidney allograft loss (61, 62). Chronic AMR appears to be less responsive to current immunosuppression compared to T cell mediated rejection (63). As a result, there is intense interest in understanding the detailed mechanisms of B cell memory generation, antibody production and plasma cell persistence. Therapies for AMR include antibody removal with plasmapheresis, B-cell depletion with agents such as rituximab, or targeting memory B cells with proteasome inhibitors such as bortezomib.

While *de novo* generation of DSAs and subsequent AMR can be devastating to a kidney allograft, the immunologic process of antibody production directed against pathogens is protective and can be lifesaving. Vaccination is a means by which both cellular and humoral memory responses can be induced to protect against life threatening pathogens. We measure vaccine responses with serologic testing however, that does not take into consideration the essential cellular components i.e., T and B lymphocytes mentioned above. While immunosuppressive therapies can successfully prevent graft rejection, they are problematic in that they prevent optimal vaccine responses. Ultimately, the goal is to fully vaccinate transplant recipients prior to transplantation in order to optimize a response before exposure to an immunosuppressive regimen. In this regard, the coronavirus disease of 2019 (COVID-19) pandemic has posed novel challenges to the transplant community.

## Vaccinations in transplant recipients

The introduction of routine childhood immunizations has saved lives (64). Many vaccine preventable diseases have either been irradicated or reduced in frequency in the general population due to national vaccine efforts. In the US, the proportion of unvaccinated children remains low (< 1%), and thus the benefits of herd immunity can protect pediatric solid organ transplant (SOT) recipients (65). The American Society of Transplantation (AST) recommends that pediatric SOT candidates be immunized prior to transplantation (66). Although it is better to vaccinate patients prior to transplant, vaccination rates in adult kidney transplant recipients remain low (67). There is no national policy to mandate pre-transplant vaccinations and as a result some patients are inadequately vaccinated prior to transplant (68). In some circumstances, patients under transplant evaluation may be receiving immunosuppressive therapies that could alter antibody responses. In those cases, it may be warranted to delay vaccination for weeks or even months depending on the immunosuppressive therapies for safety and efficacy reasons. Although vaccine rates among UNOS kidney transplant centers are improving (69) continued efforts to implement quality improvement measures must be ongoing.

All kidney transplant recipients should receive non-live vaccines based on the Kidney Disease Improving Global Outcomes (KDIGO) guidelines (70). This is especially true for those vaccines involved in cancer prevention (eg HPV). Transplant recipients should not receive live vaccines due to the risk of developing disease from the vaccine strain (66, 70, 71). Those on the transplant waitlist who receive a live virus vaccine should wait a minimum of 4 weeks before receiving a transplant. It is generally recommended to wait for 3–6 months after transplantation and until maintenance immunosuppression levels are achieved before starting vaccination in order to maximize the chances of an adequate immune response (66, 70, 71). For the same concern, vaccinations should be withheld from SOT recipients during intensified immunosuppression, such as 2–6 months after treatment of acute rejection episodes (71). However, in the setting of a global pandemic, such as with COVID-19, vaccination, albeit suboptimal, offers the possibility of decreasing mortality and morbidity of viral infection.

## SARS CoV-2 mRNA vaccination

Balancing enough immunosuppression to block the alloimmune response while permitting antigen specific immune responses to vaccines is a tightrope walk. Severe acute respiratory syndrome coronavirus 2 (SARS-CoV-2) mRNA vaccination is quite effective in a healthy general population however, lower rates of seroconversion ranging between 30-56% are seen in adult and pediatric kidney transplant recipients (72–77). The mechanisms by which immunosuppression might alter lymphocyte function in this population remains unclear. An additional wrinkle in the plot is the risk of inducing an acute rejection episode due to the heterogeneity of the immune response and possible cross reactivity (78, 79).

**Figure 1:**
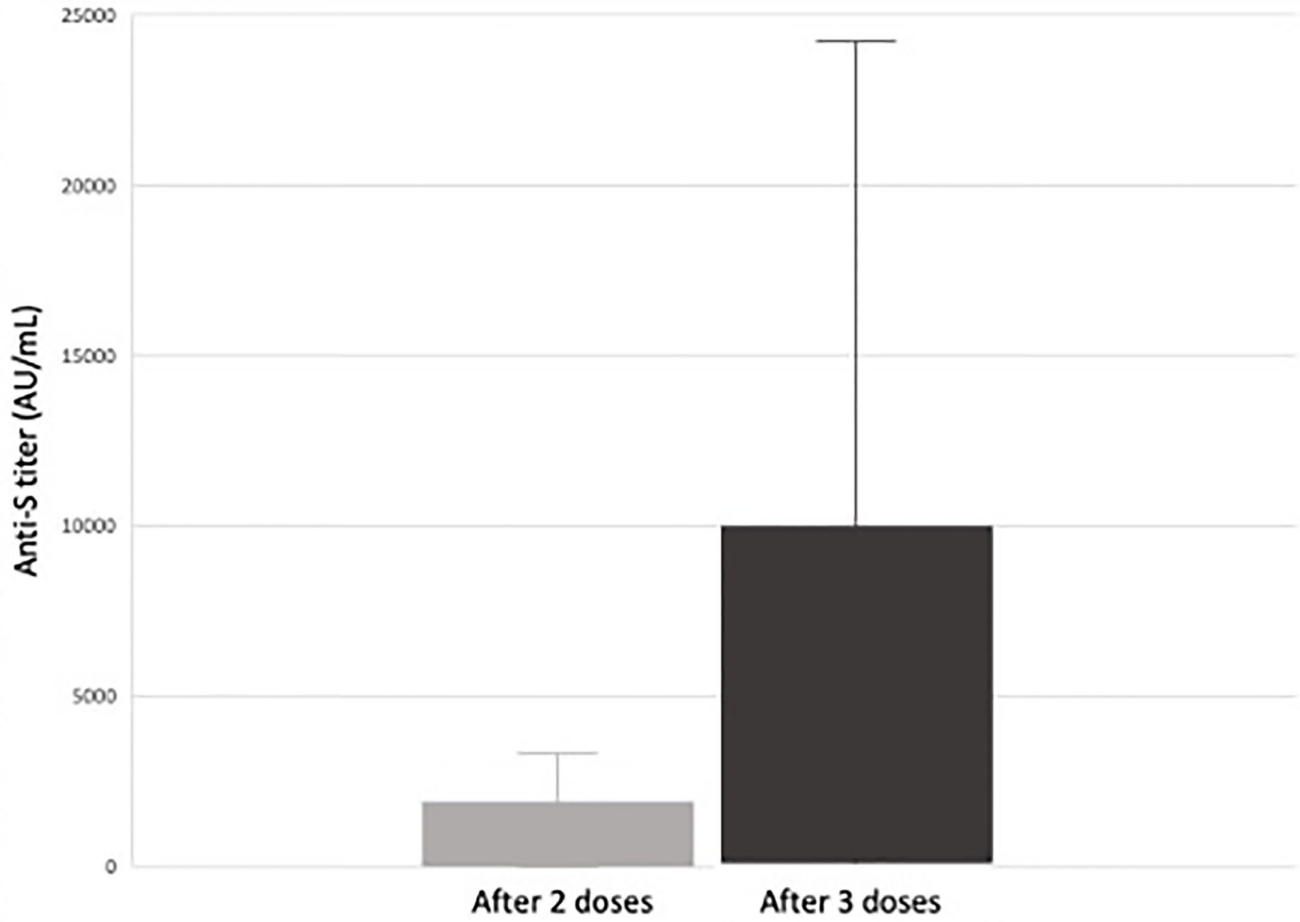
Median response and range of anti-S antibody titers (AU/mL) in response to SARS-CoV-2 vaccination in pediatric and adolescent kidney transplant recipients (80). Of the 26 pediatric and adolescent kidney transplant recipients who received a third mRNA SARS-CoV-2 vaccine dose, 22 (85%) seroconverted (defined as an anti-spike protein antibody titer >50 AU/mL). There was a significant increase in antibody titers between dose 2 and 3 from a median of 66 AU/mL after two doses to 881 AU/mL after three doses. In the 16 subjects responding only after a third dose, there was a significant increase in anti-S titer from 9.4 AU/mL to 682 AU/mL (*p* < 0.01) versus an increase from 4.9 AU/mL to 7.4 AU/mL (*p* = 0.3) in those who did not (80).

We looked at our single center retrospective cohort of pediatric and adolescent kidney transplant recipients (KTR) who received a two or three dose series of an mRNA SARS-CoV-2 vaccine. Forty-three pediatric and adolescent KTR received 2-doses of an mRNA SARS-CoV-2 vaccine and 30 received a third dose. Forty (93%) received BNT162b2 (Pfizer-BioNTech), two (5%) received mRNA-1273 (Moderna) vaccine and one received a mixed vaccine series. SARS-CoV-2 spike protein antibody levels measured 4-8 weeks after their second vaccine dose and again after the third vaccine dose. We found that 56% of pediatric kidney transplant recipients seroconverted following a 2-dose series. Seroconversion increased to 85% in those who received a third dose. In the 16 patients who did not seroconvert after a two-dose series, 12 (75%) seroconverted following the third dose (**Figure 1**). We did not observe any post-vaccination rejection episodes (80).

In a subset of those enrolled in a prospective study to examine the effects of immunosuppression on B-cell populations and infections, T and B lymphocyte subsets, immunoglobulin levels (IgA, IgG, IgM, and IgE), and vaccine titers (pneumococcal, tetanus, diphtheria, pertussis, and hepatitis B), and immunosuppressive medication doses (tacrolimus, mycophenolate mofetil, azathioprine, and prednisone) were evaluated prior to vaccination and at 6-month intervals.

**Figure 2:**
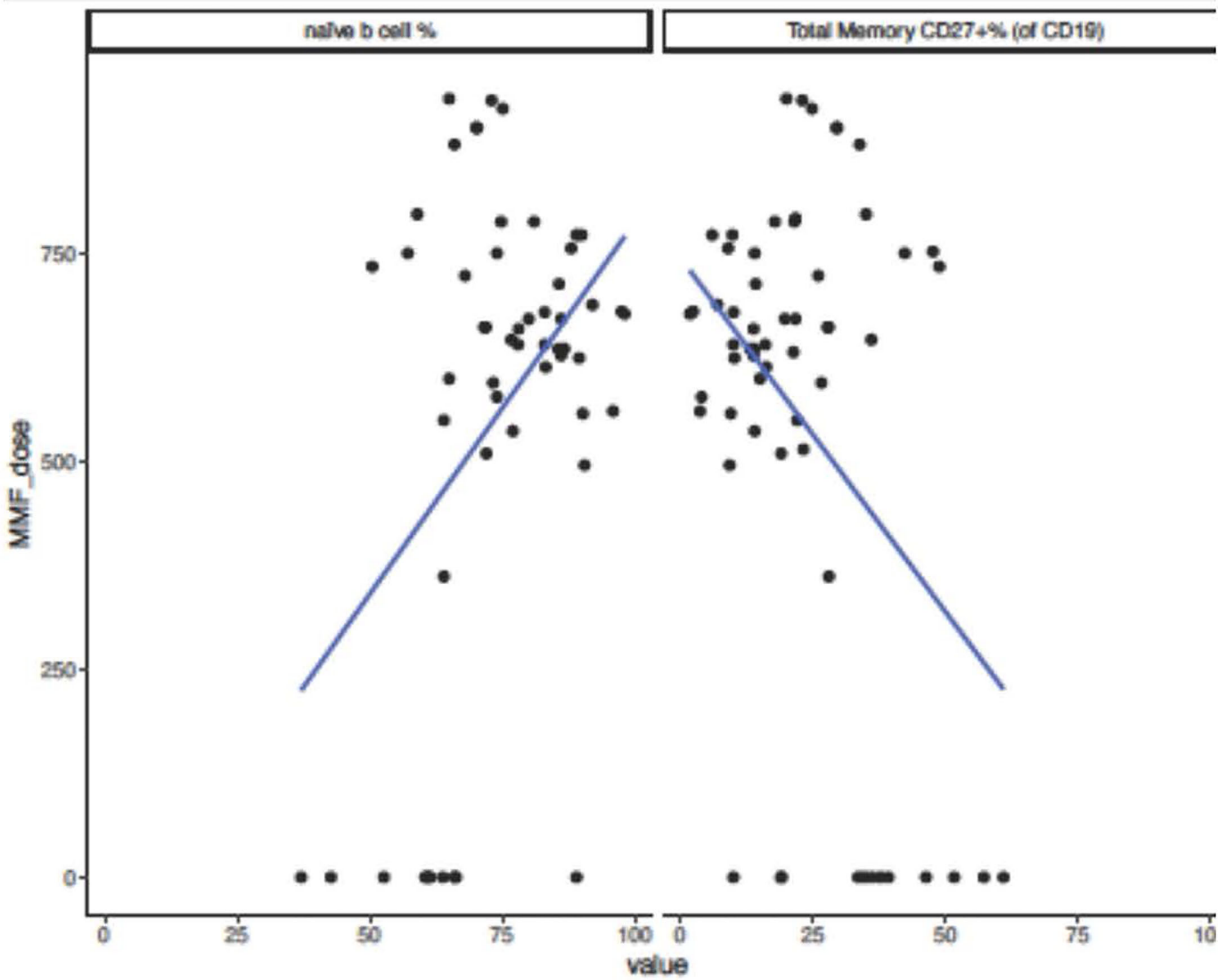
Naïve and Memory B-cell percentages and MMF dose in pediatric and adolescent kidney transplant recipients after SARS-CoV-2 mRNA vaccination. Subjects who did not seroconvert following SARS-CoV-2 mRNA vaccination showed a trend toward decreased total memory B-cell percentage (β=-12.54, p=0.080) and increased naïve B-cell percentage (β= 12.50, p=0.11). When analyzing effect of increasing MMF dose on immune parameters and increase in MMF dosing of 1 unit correlated to an increase in naïve B-cell percentage (β=0.016, p=0.0032) and decrease in total memory B-cell percentage (β=-0.016, p=0.0034).

No significant difference in immunoglobulin levels, T cell populations, or vaccine titers was observed. There was a trend toward lack of seroconversion with higher doses of mycophenolate mofetil (MMF), at 91 mg/m2/day median difference (p=0.06). All patients on azathioprine instead of MMF seroconverted. Those who did not seroconvert had lower hemoglobin levels (β=-1.30, p=0.009) and lower platelet count (β=-56.00, p=0.057). Non-responders to the vaccine showed a trend toward increased naïve B cell percentage (β= 12.50, p=0.11) and decreased total memory B cell percentage (β=-12.54, p=0.080). Increasing MMF dosage was associated with an increase in naïve B-cell percentage (β=0.016, p=0.0032) decrease in total memory B cell percentage (β=-0.016, p=0.0034) (**Figure 2**), and decreased in IgG level (β=-0.35, p=0.012).

Disruption in B cell population is likely due to immunosuppression and associated with the use of MMF. Non-responders showed non-significant trends toward high MMF dosage, increased naïve B-cell percentage, and decreased total memory B cell percentage. Trend toward decreased hemoglobin levels and normal red blood cell mean cell volume (MCV) supports that anemia could be due to bone marrow suppression caused by MMF. Future studies will need to investigate whether the defect is at the level of T cell activation or T cell help. Work in adult kidney transplant recipients has demonstrated additional vaccine doses can induce a functional maturation of vaccine-reactive T cells with significantly higher frequencies of IL-2 and IL-4 secreting and polyfunctional T cells being seen after a third dose (81).

Despite immunosuppression, the underlying immunologic machinery has the potential to “break through” after repeated exposure and stimuli. Additional SARS-CoV-2 mRNA vaccine doses are safe in kidney transplant recipients but may be necessary to overcome the iatrogenic suppression of T-cell proliferation and disruption of B-cell populations to optimize a humoral response. However, the risk of causing enough inflammation to promote acute rejection of the allograft remains a potential concern that merits ongoing observation.

## Author contributions

CC manuscript writing, data collection and analysis, LL data collection, CA statistical analysis, BG study design and analysis, EI manuscript writing, study design and analysis. All authors contributed to the article and approved the submitted version.

## Conflict of interest

The authors declare that the research was conducted in the absence of any commercial or financial relationships that could be construed as a potential conflict of interest.

## Publisher’s note

All claims expressed in this article are solely those of the authors and do not necessarily represent those of their affiliated organizations, or those of the publisher, the editors and the reviewers. Any product that may be evaluated in this article, or claim that may be made by its manufacturer, is not guaranteed or endorsed by the publisher.
